# Bioprospecting for Novel Halophilic and Halotolerant Sources of Hydrolytic Enzymes in Brackish, Saline and Hypersaline Lakes of Romania

**DOI:** 10.3390/microorganisms8121903

**Published:** 2020-11-30

**Authors:** Robert Ruginescu, Ioana Gomoiu, Octavian Popescu, Roxana Cojoc, Simona Neagu, Ioana Lucaci, Costin Batrinescu-Moteau, Madalin Enache

**Affiliations:** 1 Department of Microbiology, Institute of Biology Bucharest of the Romanian Academy, 296 Splaiul Independentei, P.O. Box 56-53, 060031 Bucharest, Romania; ioana.gomoiu@ibiol.ro (I.G.); opopescu.ubbcluj@gmail.com (O.P.); roxana.cojoc@ibiol.ro (R.C.); simona.neagu@ibiol.ro (S.N.); ioana.lucaci@ibiol.ro (I.L.); costinbatrinescu@yahoo.com (C.B.-M.); madalin.enache@ibiol.ro (M.E.); 2 Molecular Biology Center, Institute of Interdisciplinary Research in Bio-Nano-Sciences, Babes-Bolyai-University, 42 Treboniu Laurian St., 400271 Cluj-Napoca, Romania

**Keywords:** halophiles, halotolerant bacteria, halophilic archaea, hypersaline lakes, extreme environments, extremozymes, hydrolytic enzymes, extracellular hydrolases, salt-tolerant enzymes

## Abstract

Halophilic and halotolerant microorganisms represent promising sources of salt-tolerant enzymes that could be used in various biotechnological processes where high salt concentrations would otherwise inhibit enzymatic transformations. Considering the current need for more efficient biocatalysts, the present study aimed to explore the microbial diversity of five under- or uninvestigated salty lakes in Romania for novel sources of hydrolytic enzymes. Bacteria, archaea and fungi were obtained by culture-based approaches and screened for the production of six hydrolases (protease, lipase, amylase, cellulase, xylanase and pectinase) using agar plate-based assays. Moreover, the phylogeny of bacterial and archaeal isolates was studied through molecular methods. From a total of 244 microbial isolates, 182 (74.6%) were represented by bacteria, 22 (9%) by archaea, and 40 (16.4%) by fungi. While most bacteria synthesized protease and lipase, the most frequent hydrolase produced by fungi was pectinase. The archaeal isolates had limited hydrolytic activity, being able to produce only amylase and cellulase. Among the taxonomically identified isolates, the best hydrolytic activities were observed in halotolerant bacteria belonging to the genus *Bacillus* and in extremely halophilic archaea of the genera *Haloterrigena* and *Halostagnicola*. Therefore, the present study highlights that the investigated lakes harbor various promising species of microorganisms able to produce industrially valuable enzymes.

## 1. Introduction

Halophiles are a heterogeneous group of extremophilic organisms able to survive and even thrive in highly saline environments, unfavorable for the existence of most life forms known to us [1]. They can be found in all three domains of life (i.e., Bacteria, Archaea and Eukarya) and are typically categorized on the basis of their salt requirement and tolerance. The predominant inhabitants of hypersaline niches are generally represented by extremely and moderately halophilic Archaea and Bacteria. The extreme halophiles par excellence are the archaeal representatives of the class Halobacteria, most of which show optimal growth in media containing 2.5 to 5.2 M NaCl, although some extremely halophilic Bacteria (e.g., genus *Salinibacter*) have been described as well [2,3]. Moderately halophilic (growing best at 0.5–2.5 M NaCl) and halotolerant bacteria (growing in the absence as well as in the presence of relatively high salt concentrations) are spread over a large number of phylogenetic branches, most species being grouped in the Proteobacteria (Gamma- and Alphaproteobacteria), Firmicutes and Actinobacteria [4]. Within the domain Eukarya, halophily is less widespread, the best salt-adapted eukaryotes being represented by few species of green algae (e.g., *Dunaliella*), yeasts (e.g., *Hortaea werneckii*), filamentous fungi (e.g., *Wallemia ichthyophaga*) and protozoa (e.g., *Halocafeteria seosinensis*) [5].

The great diversity of halophiles is expressed not only at the phylogenetic level but also at the physiological level; most microbial processes of energy generation that occur at low salt concentrations having been identified up to considerably high salinities. In this regard, the metabolic diversity of halophilic microorganisms includes oxygenic and anoxygenic phototrophs, aerobic chemo-organoheterotrophs and chemolithoautotrophs, fermenters, denitrifiers, sulfate reducers, methanogens and acetogens [1,2]. Moreover, considerable diversity also exists in the mechanisms these microorganisms use to cope with the osmotic pressure exerted by the high salt concentration of the surrounding medium [5].

The heterogeneity within communities of halophilic microorganisms is a reflection of their ability of adaptation to a wide range of habitats that are characterized by different and often changing physicochemical conditions (overall salinity and ionic composition, pH, temperature, oxygen availability, nutrient status, etc.). Many salty environments, including inland lakes, coastal salterns, soils, marshes, deep hypersaline anoxic basins, salted foods, salt rocks, leaves of some plants that grow in very salty soils, salt glands and nasal cavities of some animals and even surfaces of archaeological monuments and wall paintings have been described as being appropriate to sustain the survival of halophiles [2]. Of these, natural hypersaline lakes of oceanic (thalassohaline) and non-oceanic (athalassohaline) origin, as well as solar salterns, have been most explored in terms of their microbiota using both culture-dependent and culture-independent techniques [6,7].

Over recent decades, halophiles have been studied mainly for their possible applications in biotechnological and environmental processes. The two most successful industrial processes involving halophilic microorganisms are the production of β-carotene by the unicellular alga *Dunaliella* and the production of ectoine using various species of moderately halophilic bacteria [8,9]. Moreover, many other products synthesized by halophiles (e.g., bacteriorhodopsin, enzymes, polysaccharides, polyhydroxyalkanoates, biosurfactants, antimicrobials) or processes performed by them (e.g., degradation of toxic compounds) have found different actual or potential commercial uses [10,11,12,13]. In comparison to mesophilic enzymes, halophilic and halotolerant counterparts are active over a broad range of salinities and could, therefore, be used in many challenging industrial processes where high salt concentrations would otherwise inhibit enzymatic transformations. These salt-adapted enzymes are characterized by an excess of acidic amino acids at the protein surface and by a general decrease in hydrophobic amino acid frequency [14,15]. Several halophilic enzymes, including glycosidases, proteases and lipases have been purified and characterized in recent years, providing promising opportunities particularly for biofuel production [16], food processing [17] and biodegradation of organic pollutants [18,19].

In Romania, saline environments represented mainly by salt mines and athalassohaline lakes are widely spread. The latter are highly diverse in terms of ionic composition and overall salinity and generally harbor large deposits of organic- and mineral-rich sediments, which are of particular utility in balneotherapy [20]. The southeastern region of the country (Romanian Plain) is characterized by the presence of five major salty lakes (i.e., Amara, Balta Alba, Caineni Bai, Movila Miresii and Braila Salt Lake) with salinities ranging from brackish (<36 g·L^−1^, local seawater) to hypersaline (>50 g·L^−1^) depending on rainfall, water evaporation rate and the basin substrate. The degree of mineralization of Lake Amara has varied over recent years between 6.5 and 31 g·L^−1^, the dominant ion being sulfate, followed by chloride, sodium and small amounts of magnesium [21,22,23]. The salinity of the other four lakes has also fluctuated between 8.9 and 16 g·L^−1^ in Lake Balta Alba [21,24], 29 and 45 g·L^−1^ in Lake Caineni Bai [21,25], 57 and 150 g·L^−1^ in Movila Miresii Salt Lake [26,27] and 111 and 317 g·L^−1^ in Braila Salt Lake [22,24], the predominant ions being chloride and sodium, followed by sulfate and considerably lower quantities of magnesium.

The biota of these ecosystems has been characterized particularly in terms of phyto- and zooplankton composition [24,28,29], but little or no information about their bacterial, archaeal and fungal communities is currently available [30,31,32]. Hence, these environments remain largely unexplored reservoirs of microorganisms potentially capable of producing novel bioactive compounds and industrially valuable molecules. In the present study, we aimed to (1) cultivate and isolate aerobic, chemo-organoheterotrophic, halophilic/halotolerant bacteria, archaea and fungi from five saline lakes in Romania; (2) determine their salt requirement/tolerance and phylogeny; and (3) assess whether they produce various extracellular hydrolases whose property of being stable and active at high salt concentrations may be useful in biotechnology.

## 2. Materials and Methods

### 2.1. Sampling and Measurement of Physicochemical Parameters

**Figure 1:**
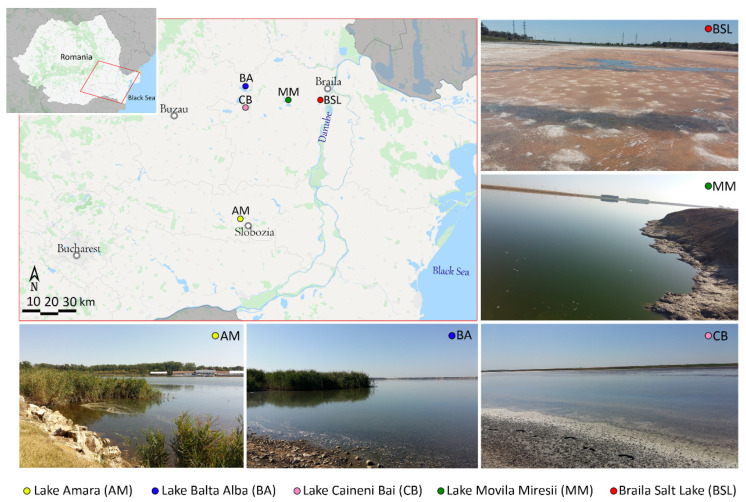
Geographic locations and overview photos of the five studied lakes. Three sites per lake were sampled: Lake Amara (AM) (44°36′20.9″ N, 27°19′39.6″ E; 44°36′23.8″ N, 27°19′35.9″ E; 44°36′23.0″ N, 27°19′14.9″ E), Lake Balta Alba (BA) (45°17′38.1″ N, 27°20′54.1″ E; 45°17′37.1″ N, 27°20′55.6″ E; 45°17′40.7″ N, 27°20′51.8″ E), Lake Caineni Bai (CB) (45°11′00.3″ N, 27°20′01.4″ E; 45°11′00.7″ N, 27°19′59.2″ E; 45°10′54.8″ N, 27°19′28.2″ E), Movila Miresii Salt Lake (MM) (45°13′16.0″ N, 27°38′25.8″ E; 45°13′15.4″ N, 27°38′31.6″ E; 45°13′16.0″ N, 27°38′20.1″ E), Braila Salt Lake (BSL) (45°12′57.6″ N, 27°54′38.4″ E; 45°12′58.8″ N, 27°54′37.7″ E; 45°12′58.4″ N, 27°54′40.3″ E).

Water and sediment samples were collected from five salty lakes located in the Romanian Plain: Lake Amara (AM), Lake Balta Alba (BA), Lake Caineni Bai (CB), Movila Miresii Salt Lake (MM) and Braila Salt Lake (BSL) (Figure 1), during a two-day field trip in August 2019. At each of the five locations, three sampling sites positioned at 1–15 m from the shore and at 0.25–0.8 m below the water surface were randomly selected, totaling three water samples and three sediment samples per lake. GPS coordinates were recorded from each sampling site (Figure 1). Sterile 250 mL glass bottles and 50 mL plastic tubes were used to collect water and sediments, respectively. Samples were transported to the laboratory in thermal bags at about 4 °C and processed within 48 h after collection.

Physicochemical parameters (temperature, pH, dissolved oxygen, oxidation-reduction potential, salinity, electrical conductivity) were measured in situ with a portable multiparameter instrument for water analysis (Hanna HI98194). Moreover, considering that chloride was the dominant ion in almost all the investigated environments (except AM), its concentration was determined in the laboratory by titration (Mohr’s method) with AgNO_3_ [33].

### 2.2. Cultivation and Abundance Estimation of Halophilic and Halotolerant Microorganisms

In order to isolate halophilic and halotolerant microorganisms and estimate their cultivable fraction, water and sediment samples were serially diluted and plated onto two different growth media (HM [34] and JCM 168 [35]) with salinities ranging from 5% to about 22% (*w*/*v*). To avoid the stress effect of low salinity conditions on halophilic systems [36], dilutions were made in sterile saline solutions containing 5%, 10% or 20% (*w*/*v*) NaCl. Aliquots (1 mL) of each decimal dilution (10^−1^, 10^−2^ and 10^−3^) were inoculated in triplicate by pour-plating [37]. The plates designated for the growth of halotolerant and moderately halophilic bacteria and fungi (i.e., HM containing 5% and 10% salts) were incubated at 30 °C for 10 days (in the case of bacteria) or at 24 °C for 30 days (in the case of fungi). The plates designated for the growth of extremely halophilic bacteria and archaea (i.e., HM containing 20% salts and JCM 168) were incubated at 37 °C for 40 days in sealed polyethylene bags. Following incubation, colonies were counted, and results were expressed as colony-forming units (CFU) per 1 mL of water or 1 g of wet sediment. Moreover, colonies that displayed relatively distinct morphologies were purified by streak-plating [37] onto the same growth medium used for the initial cultivation.

The compositions of the growth media used in the present study were as follows (in g·L^−1^). HM growth medium [34]: NaCl (31, 81, or 181), MgCl_2_∙6H_2_O (7), MgSO_4_∙7H_2_O (9.6), CaCl_2_∙2H_2_O (0.36), KCl (2), NaHCO_3_ (0.06), NaBr (0.026), glucose (1), proteose-peptone (5), yeast extract (10) and agar (20). JCM 168 growth medium [35]: casamino acids (5), yeast extract (5), sodium glutamate (1), trisodium citrate (3), MgSO_4_∙7H_2_O (20), KCl (2), NaCl (200), FeCl_2_∙4H_2_O (0.036), MnCl_2_∙4H_2_O (traces) and agar (20). The pH of the culture media was adjusted to 7.2 before autoclaving.

### 2.3. Halophily and Halotolerance Assessment of the Isolates

In order to evaluate salt requirement and tolerance of the microbial isolates, solid HM and JCM 168 growth media containing different NaCl concentrations (0, 0.5, 1, 2, 3, 3.5, 4 and 4.5 M) were used. Each isolate was spot-inoculated onto the surface of the appropriate culture media using fresh solid inoculum. Growth was monitored daily and considered positive when it was visible after 14 days of incubation at 30 °C (for fast-growing bacteria), after 30 days of incubation at 24 °C (in the case of fungi) or after 40 days of incubation at 37 °C (for slow-growing archaea). Moreover, the salt concentrations at which growth appeared first were considered optimal. The isolates were categorized as halotolerant, moderate halophiles or extreme halophiles according to the classification scheme proposed by Kushner [38].

### 2.4. Screening for Extracellular Enzyme Activities

The capability of halotolerant and moderately halophilic bacteria and fungi to produce hydrolytic enzymes (protease, lipase, amylase, cellulase, xylanase and pectinase) was qualitatively assayed on modified HM media containing 10% (*w*/*v*) salts and one of the following substrates of interest (g·L^−1^): casein (1), Tween-80 (1), starch (1), carboxymethyl cellulose (CMC) (0.5), xylan (1) or pectin (1). Glucose and proteose-peptone were removed from the composition of these assay media. Each microbial isolate was spot-inoculated onto the surface of agar plates using fresh solid inoculum and incubated at 30 °C for 14 days. Subsequently, hydrolytic activities against casein, starch, pectin, CMC and xylan were indicated by a clear zone around the colonies after flooding the plates with 1 N HCl (in the case of casein), 0.3% I_2_–0.6% KI solution (in the cases of starch and pectin) or 0.1% Congo red solution (in the cases of CMC and xylan). Lipolytic activity, on the other hand, was indicated by an opaque halo around the colonies due to the precipitation of fatty acids resulted from Tween-80 hydrolysis [39,40].

Extremely halophilic archaea and bacteria were screened for hydrolytic enzyme production on modified JCM 168 media containing 3 M NaCl and the same substrates mentioned above. It is important to note that casamino acids were removed from the composition of these assay media and the amount of yeast extract was reduced to only 1 g·L^−1^ [41]. The inoculation and interpretation of results were carried out as described above, while incubation was performed at 37 °C for 30–45 days.

All the screening experiments were conducted in duplicate, and results were expressed qualitatively as levels of enzyme activities (LEA) using the formula: LEA = diameter of the hydrolysis zone divided by the diameter of the microbial colony (in millimeters) [40,42]. Consequently, the microbial isolates were categorized as having high (LEA > 3), medium (LEA 2–3), low (LEA < 2) or no hydrolytic activities.

### 2.5. DNA Extraction and PCR Amplification of the 16S rRNA Gene

Bacterial and archaeal isolates with distinct phenotypic characteristics (colony morphology, salt tolerance/requirement, extracellular hydrolytic enzyme profiles) were selected for molecular identification by PCR amplification and sequencing of the 16S rRNA gene. To this end, each isolate was grown in the appropriate liquid medium (HM or JCM 168) under agitation (160 rpm) for 24–48 h at 30 °C (for fast-growing bacteria), or for 20–30 days at 37 °C (for slow-growing archaea). Cells contained in 1 mL of culture were harvested in a microcentrifuge tube by centrifuging for 10 min at 5000× *g*. Genomic DNA was extracted using a DNeasy Blood & Tissue Kit (Qiagen, Hilden, Germany) following the standard protocol for bacteria. In order to ensure efficient isolation of DNA from difficult-to-lyse Gram-positive bacteria, harvested cells were preincubated (at 37 °C for 2–3 h) with an enzymatic lysis buffer containing Tris∙HCl (20 mM), EDTA (2 mM), Triton X-100 (1.2%, *v*/*v*) and lysozyme (20 mg·mL^−1^) before DNA purification. Concentration and purity of genomic DNA were checked using a BioDrop DUO UV/VIS spectrophotometer.

PCR amplification of the 16S rRNA gene was carried out in a 50 μL final reaction volume containing 1× Mango Master Mix (Bioline), 0.2 μM of each primer, 50–250 ng DNA template and water. Primers 27F (AGAGTTTGATCMTGGCTCAG) and 1492R (ACGGYTACCTTGTTACGACTT) [43] were used to amplify bacterial DNA, while primers 20F (TCCGGTTGATCCTGCCG) and 1530R (GGAGGTGATCCAGCCG) [41] were used for the amplification of archaeal DNA. PCR reactions were performed using a Mastercycler Pro S Thermal Cycler (Eppendorf) under the following conditions: 3 min denaturation at 95 °C, 35 cycles of 1 min denaturation at 95 °C, 1 min annealing at 57 (for 27F/1492R) or 50 °C (for 20F/1530R), 90 s extension at 72 °C and a final extension step of 5 min at 72 °C. Amplicons were checked on agarose gel (1%, *w*/*v*) and then were purified using a QIAquick PCR Purification Kit (Qiagen).

### 2.6. Gene Sequencing and Phylogenetic Analysis

Purified amplicons were directly sequenced by a commercial sequencing service provider (Macrogen Europe B.V.) using the forward primers 27F (for Bacteria) and 20F (for Archaea). The obtained chromatograms were analyzed using the CodonCode Aligner software (version 9.0.1), and sequencing inaccuracies were manually edited. The resulting sequences were compared to known sequences available in the NCBI public database using the BLASTN algorithm [44]. Subsequently, the 16S rRNA gene sequences of related reference strains were downloaded from NCBI and used for multiple sequence alignments in the MEGA X software (CLUSTALW algorithm). Finally, the phylogenetic trees were constructed in MEGA X from the resulting alignments, using the Neighbor-joining method and the Tamura-Nei model [45].

### 2.7. Nucleotide Sequence Accession Numbers

The partial 16S rRNA gene sequences of the bacterial and archaeal isolates were deposited in GenBank (NCBI) under the accession numbers MW036374–MW036445 and MW052695–MW052707, respectively.

## 3. Results

### 3.1. Sampling Sites Description

The five sampled lakes are highly dynamic ecosystems whose water chemistry and temperature fluctuate seasonally depending on climate conditions (i.e., rainfall, solar radiation intensity and water evaporation rate) [21,22,23,24,25,26,27]. The physicochemical parameters measured at the time of sampling (August 2019) are presented in Table 1. Based on water salinity, the investigated environments were of three types: brackish (AM and BA), saline (CB) and hypersaline (MM and BSL). Their alkaline pH (8–10) was slightly higher than previously reported [21,22,23,24,25,26,27], and the relatively elevated water temperatures (23–39 °C) were due to the shallow depths of these water bodies during the dry periods. Dissolved oxygen (DO) concentrations decreased with the increase in salt levels and were generally low (0.3–4.6 mg·L^−1^), with the only exception of AM where higher amounts (10–12.8 mg·L^−1^) were measured. Hypoxic conditions (DO < 2 mg·L^−1^) were encountered only in BSL, where the combined effects of high salinity and elevated temperature resulted in a reduction in oxygen solubility. Moreover, the low levels of DO, together with the negative values of the oxidation-reduction potential (ORP) measured in BA, CB, MM and BSL, could have been related to an increased activity of microbial decomposers in the benthic layer of these shallow lakes [46].

In addition to physicochemical differences, the sampled environments showed some color distinctions that could suggest the dominant components of their biological communities. While AM, BA and CB sites showed to be turbid and brown, most probably due to high amounts of organic-rich sediments, MM water was characterized by a green color (Figure 1), which could be explained by the chlorophyll contained in the abundantly present algae [28]. On the other hand, both the red water and the reddish salt crust observed in BSL (Figure 1) could have derived their color from dense communities of carotenoid-rich microorganisms. In this regard, the members of the class Halobacteria, the bacterium *Salinibacter* and the alga *Dunaliella* are considered to be the main sources of carotenoids in hypersaline niches [47].

### 3.2. Abundance of Cultured Halophilic and Halotolerant Microorganisms

**Figure 2:**
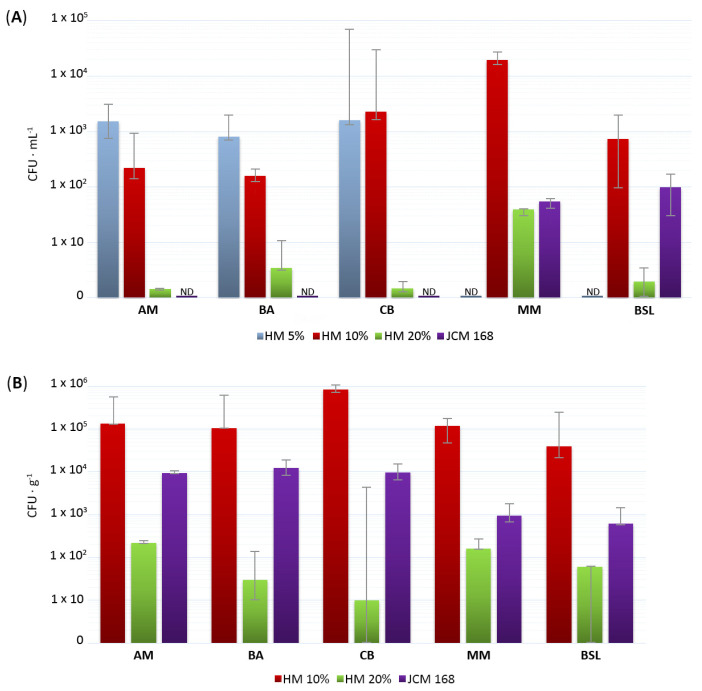
The abundance, expressed as colony-forming units (CFU) per 1 mL of water (**A**) or 1 g of wet sediment (**B**), of the cultured fractions of halophilic/halotolerant microbial communities inhabiting the five investigated lakes. Bars show the differences between the three sampling sites. ND = Not Determined.

The cultured fractions of the microbial communities inhabiting the five investigated environments varied considerably in abundance not only from one ecosystem to another but also between the different sites of the same lake (Figure 2). The highest microbial densities were generally obtained on HM media containing 10% salts, in which case the CFUs ranged from 9.4 × 10 (in BSL) to about 3 × 10^4^ (in CB and MM) per mL of water (Figure 2A) and from 2.2 × 10^4^ (in BSL) to about 1 × 10^6^ (in CB) per gram of wet sediment (Figure 2B). Another general observation was that the CFUs decreased with increasing NaCl concentration in the growth media composition, suggesting that most of the cultured microorganisms could be halotolerant or moderately halophilic. Moreover, although HM 20% and JCM 168 media contained similar salt concentrations, the latter showed to be much more suitable for the growth of extremely halophilic archaea and bacteria than the former.

### 3.3. Halophily and Halotolerance of the Isolates

A total of 244 microbial isolates (182 bacteria, 22 archaea and 40 fungi) from the five sampled lakes were obtained in pure cultures and tested for the ability to grow at different salt concentrations. The majority (141 isolates, 57.8%) grew between 0 and 2 M NaCl (optimally at 0–1 M), and thus they were categorized as halotolerant. However, some of these isolates, particularly those recovered from the hypersaline lake BSL, were able to grow slowly (>10 days of incubation) up to 3–3.5 M NaCl and, therefore, they were considered extremely halotolerant. A significantly smaller fraction of isolates was constituted by moderately (75 isolates, 30.7%) and extremely halophilic (28 isolates, 11.5%) microorganisms, whose growth has shown to be dependent on relatively high salt concentrations. While most of the former grew in the NaCl range of 0.5–3 M (optimally at 0.5–1 M), the majority of the latter grew between 2 and 4.5 M NaCl (optimally at 2–3.5 M).

**Figure 3:**
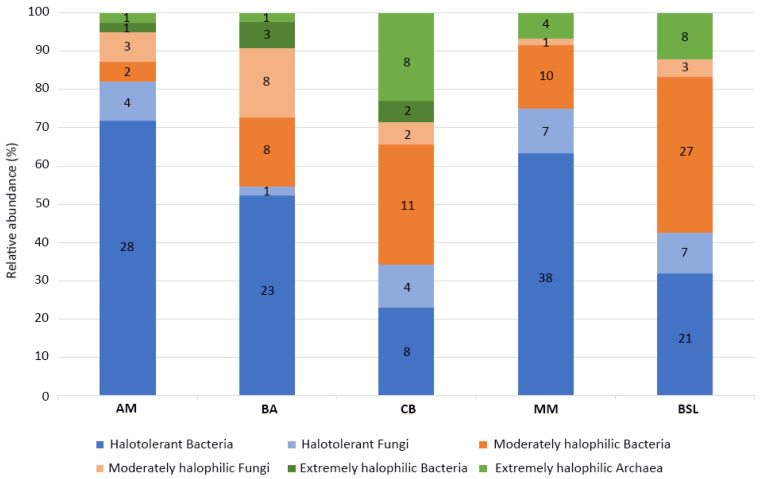
Relative abundances of halotolerant and halophilic microorganisms isolated from the five investigated lakes. The numbers in the bar graphs indicate the number of isolates.

In four of the five investigated lakes (i.e., AM, BA, CB and BSL), it was observed that the number of cultured halotolerant isolates decreased in favor of moderately and extremely halophilic ones as the salinity of the lakes increased (Figure 3). Nevertheless, this tendency was not observed in MM, where most of the cultured isolates did not show to be salt-dependent despite the hypersaline conditions encountered at the time of sampling. Moreover, extremely halophilic bacteria and archaea were isolated, albeit in low numbers, even from the brackish lakes AM and BA whose salinities are—according to the classification scheme of halophilic microorganisms [38]—unsuitable for the growth of such extremophiles (Figure 3).

### 3.4. Production of Extracellular Hydrolytic Enzymes

**Figure 4:**
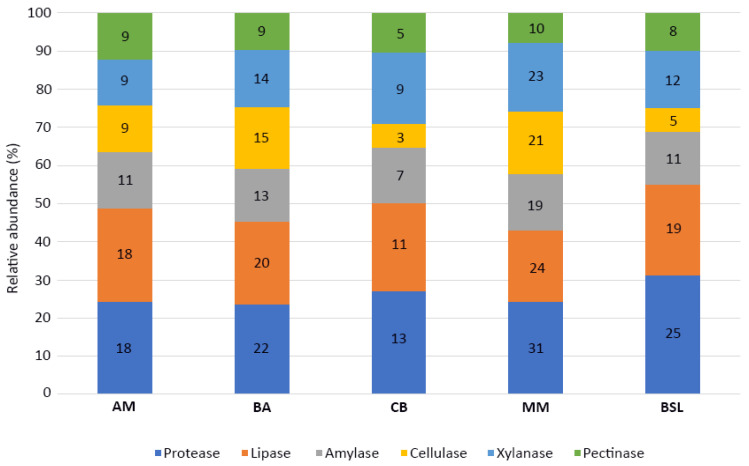
Relative abundances of microbial hydrolase producers recovered from the five investigated lakes. The numbers in the bar graphs indicate the number of isolates that produced a particular enzyme.

All 244 microbial isolates were screened for the ability to synthesize hydrolytic enzymes capable of degrading various types of substrates, such as proteins (i.e., casein), lipids (i.e., Tween-80) and polysaccharides (i.e., starch, CMC, xylan and pectin). While 88 isolates (36%) produced none of the six hydrolases tested, the majority (156 isolates, 64%) showed single or combined hydrolytic activities. The enzymes most frequently produced by the microorganisms cultured from each of the five salty lakes were protease and lipase (Figure 4). However, major differences in the type and number of extracellular hydrolytic activities were observed between the various bacterial, archaeal and fungal isolates.

From a total of 182 bacterial isolates, 94 (51.6%) were protease producers, 79 (43.4%) lipase producers, 53 (29%) xylanase producers, 51 (28%) amylase producers, 34 (18.7%) cellulase producers, and only 11 (6%) isolates were able to synthesize pectinase. There were no observed significant differences in the extracellular enzymatic profiles with respect to the origin and nature of samples (water or sediments) from which bacteria were cultured. Combined hydrolytic activities were detected in 81 (44.5%) bacterial isolates. Of these, seven (8.6%) presented all six hydrolases tested, eight (9.9%) produced five hydrolases, 17 (21%) produced four hydrolases, 28 (34.5%) showed three hydrolytic activities and 21 isolates (26%) were able to produce two hydrolytic enzymes. Single enzymatic activities (generally proteolytic, lipolytic or amylolytic) were identified in 35 isolates (19.2%), while 66 bacteria (36.3%) showed none of the hydrolytic activities.

In contrast to bacterial isolates, the potential of the 22 archaeal representatives to produce extracellular hydrolytic enzymes was quite limited. In this regard, the only two hydrolases produced by only a few isolates were amylase (four isolates, 18%) and cellulase (two isolates, 9%). All the other tested substrates did not represent a readily usable carbon source for the growth of these prokaryotic microorganisms.

Among the 40 fungal isolates, 30 (75%) were pectinase producers, 17 (42.5%) cellulase producers, 15 (37.5%) protease producers, 14 (35%) xylanase producers, 13 (32.5%) lipase producers and only 6 (15%) isolates produced amylase. Combinations of different hydrolytic activities were detected in 26 (65%) fungal isolates. Of these, one (3.9%) was able to synthesize all the six tested hydrolases, 11 (42.3%) presented four hydrolytic activities, seven (26.9%) showed three hydrolytic activities and seven (26.9%) produced two hydrolases. Ten isolates (25%) showed only one hydrolytic activity (generally pectinolytic or cellulolytic), while four isolates (10%) were not able to produce any extracellular hydrolase.

### 3.5. Phylogenetic Affiliation of Bacterial and Archaeal Isolates

A total of 85 prokaryotic isolates (72 bacteria and 13 archaea) from the investigated salty lakes were selected based on their phenotypic characteristics (colony morphology, salt tolerance/requirement, extracellular hydrolytic enzyme profiles) and subjected to genotypic identification by amplification and sequencing of the 16S rRNA gene. Fungal isolates, however, were not taxonomically identified, the present paper dealing only with their extracellular hydrolytic activities and salt tolerance.

**Figure 5:**
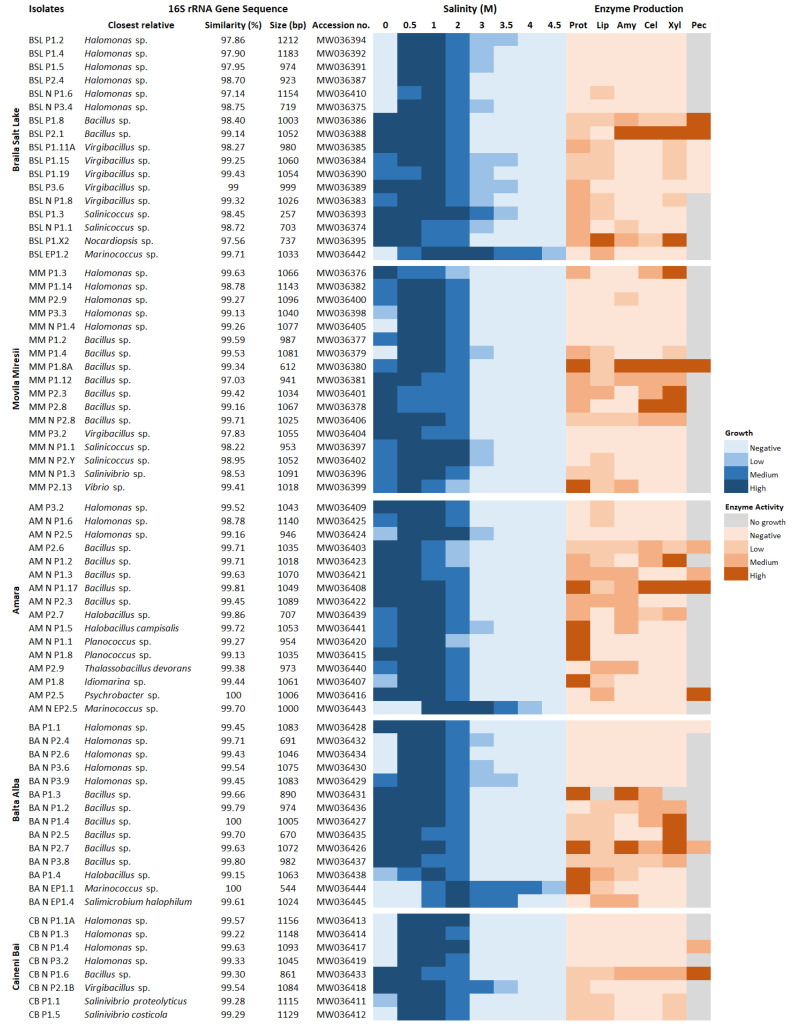
Heat maps showing the ability of the *Bacterial* isolates—taxonomically identified on the basis of the 16S rRNA gene sequence analysis—to grow at different salt concentrations and produce extracellular hydrolytic enzymes.

Comparative analysis of partial 16S rRNA gene sequences (700–1200 bp) (Figure 5) revealed that bacterial isolates fell within three phyla: Firmicutes (42 isolates, 58.3%), Proteobacteria (29 isolates, 40.3%) and Actinobacteria (one isolate, 1.4%). Among the Firmicutes genera, *Bacillus* was the most dominant (21 isolates), followed by *Virgibacillus* (seven isolates), *Salinicoccus* (four isolates), *Marinococcus* (three isolates), *Halobacillus* (three isolates), *Planococcus* (two isolates), *Thalassobacillus* (one isolate) and *Salimicrobium* (one isolate). Within the phylum Proteobacteria (Class Gammaproteobacteria), the majority of isolates were found to belong to the *Halomonas* genus (23 isolates) and only a few were related to members of the *Salinivibrio* (three isolates), *Vibrio* (one isolate), *Idiomarina* (one isolate) and *Psychrobacter* (one isolate) genera. Moreover, the only isolate belonging to the phylum Actinobacteria was closely related to representatives of the genus *Nocardiopsis*. The inferred phylogenetic trees showing the clustering of bacterial isolates with the most closely related reference strains are presented in Figure S1 (for Firmicutes) and Figure S2 (for Proteobacteria and Actinobacteria).

**Figure 6:**
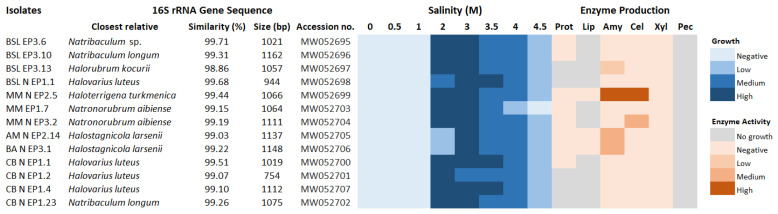
Heat maps showing the ability of the *Archaeal* isolates—taxonomically identified on the basis of the 16S rRNA gene sequence analysis—to grow at different salt concentrations and produce extracellular hydrolytic enzymes.

The archaeal isolates fell within three orders of the class Halobacteria (phylum Euryarchaeota): Halobacteriales (seven isolates), Haloferacales (one isolate) and Natrialbales (five isolates). Within the Halobacteriales order, three isolates showed a high 16S rRNA gene sequence similarity with *Natribaculum longum*, and four isolates had a high degree of homology with *Halovarius luteus*. One isolate belonging to the order Haloferacales was closely related to *Halorubrum kocurii*. The order Natrialbales, on the other hand, included two isolates related to *Halostagnicola larsenii*, one isolate related to *Haloterrigena turkmenica* and two isolates affiliated with *Natronorubrum aibiense* (Figure 6 and Figure S3).

## 4. Discussion

Over recent decades, saline and hypersaline environments have gained considerable attention from the scientific community due to their natural inhabitants, which have adapted to synthesize various biotechnologically valuable compounds such as hydrolytic enzymes [8,9,10]. Several studies performed in various areas around the world have reported the discovery of different halophilic and halotolerant microbial taxa capable of producing robust hydrolases that retain their catalytic activity over a wide range of salinities and, in some cases, even under extreme conditions of pH and temperature [16,39,40,48,49,50,51,52]. Although various such extremozymes have been described to date, there are still many unexplored saline and hypersaline environments that could harbor novel microbial strains able to produce biomolecules with favorable characteristics for biotechnological applications. In this context, in the present study, five un- or underexplored lakes in Romania with salinities ranging from brackish to hypersaline were microbiologically investigated by culture-dependent approaches in order to identify novel and more efficient producers of hydrolytic enzymes.

The microorganisms recovered from each investigated lake (i.e., AM, BA, CB, MM and BSL) were represented primarily by bacteria and secondarily by archaea and filamentous fungi (Figure 3). From a total of 244 microbial isolates, 182 (74.6%) were represented by bacteria, 22 (9%) by archaea and 40 (16.4%) by fungi. The majority of bacterial and fungal isolates were halotolerant (141 isolates, 57.8%) or moderately halophilic (75 isolates, 30.7%), and only a small fraction of the cultured microorganisms was constituted by extremely halophilic bacteria (6 isolates, 2.5%) and archaea (22 isolates, 9%) (Figure 3). A clear correlation between the halophily/halotolerance of these microorganisms and the salinity of the environments from which they were recovered was not found. In this respect, it is interesting to note that halotolerant and moderately halophilic microorganisms dominated over the extremely halophilic ones even in the hypersaline lakes MM and BSL (Figure 2). This finding could have been related to the large salinity fluctuations periodically encountered in these environments [21,22,23,24,25,26,27]. Furthermore, considering the generally accepted statement that cultivable microorganisms represent only a small fraction of microbial communities [53], the results reported in the present study could have looked very different from a metagenomic perspective.

In contrast to our results, previous culture-based investigations conducted in different hypersaline environments have reported a higher prevalence of moderately and extremely halophilic microorganisms than halotolerant species [39,52,54]. The differences are, however, understandable if considering that these habitats are characterized by higher salinities than the lakes investigated in the present work.

The screening for extracellular hydrolytic enzyme production among the 244 cultured microorganisms showed that 156 isolates (64%) were able to exhibit at least one of the six hydrolytic activities tested. The origin and nature (water or sediments) of samples from which the isolates were recovered did not influence their extracellular enzymatic profiles. In this regard, the most frequent enzymatic activities detected in each of the five lakes were proteolytic (109 isolates) and lipolytic (92 isolates) (Figure 4). However, clear distinctions between the hydrolytic abilities of bacterial, archaeal and fungal isolates were observed. While most bacteria synthesized protease (94 isolates) and lipase (79 isolates), the hydrolase most frequently produced by fungi was pectinase (30 isolates). The archaeal isolates, on the other hand, had limited hydrolytic activity, the only two enzymes produced by them being amylase (four isolates) and cellulase (two isolates). In addition, it was interesting to observe that pectin had an inhibitory effect on the growth of most bacterial and archaeal isolates, but not on fungi. The antibacterial activity of pectin against various species has been previously reported in the literature [55,56,57].

Previous studies that aimed to screen the hydrolytic potential of halophilic and/or halotolerant bacteria isolated from various aquatic environments—i.e., solar salterns in Spain [48], a hypersaline lake in Iran [39], and sea sediments from the East China Sea [58]—have shown that most isolates were able to produce amylase, lipase and protease. These results are partially in line with those reported in the present paper. However, very different results were reported in another two studies carried out in hypersaline habitats in Morocco [52] and Iraq [49]. While the former [52] reported the prevalence of bacteria producing cellulase and pectinase, the latter [49] showed that the enzymes most frequently produced by bacterial isolates were pectinase, amylase and lipase. On the other hand, halophilic archaea recovered from various Algerian hypersaline habitats have shown higher hydrolytic abilities compared to the isolates reported in the present paper, most of them being able to produce esterase, inulinase and gelatinase [40] or esterase, protease and amylase [50]. In addition, halotolerant and halophilic fungi isolated from hypersaline environments have been reported, albeit only in a few studies [51,59], as good producers of protease, cellulase, amylase, lipase and chitinase.

The taxonomic identification of 72 bacterial isolates revealed that most of them were related to species of the genera *Halomonas* (23 isolates) and *Bacillus* (21 isolates). All of the five investigated lakes harbored representatives of these two taxonomic groups, this observation being in accordance with other previous studies performed in various saline and hypersaline environments around the world [39,48,49,60]. The *Bacillus* isolates were generally halotolerant and exhibited higher hydrolytic activities compared to the other cultured taxa. In this regard, they produced combinations of three or more enzymes, mainly protease (19 isolates), lipase (17 isolates), xylanase (17 isolates), cellulase (16 isolates) and amylase (14 isolates). Moreover, six isolates (BSL P1.8, MM P1.8A, AM P2.6, AM N P1.17, BA N P2.7, CB N P1.6) were able to degrade all six substrates tested (Figure 5). Members of the genus *Bacillus* are well known for their ability to synthesize bioactive molecules, and several mesophilic and alkaliphilic strains are currently used for the industrial production of enzymes [61,62]. However, to the best of our knowledge, halotolerant or halophilic strains are not industrially exploited, although their salt-tolerant metabolites may be more efficient in certain industrial processes than their mesophilic counterparts [16,17,18,19].

Although numerous moderately halophilic species belonging to the genus *Halomonas* have been reported in previous studies as good enzyme producers [39,48], most of the isolates screened in the present research did not show any hydrolytic activity (Figure 5). Other taxa that were characterized by great hydrolytic potentials were generally halotolerant. For instance, some isolates (AM P2.7, AM N P1.5, AM N P1.1, AM N P1.8, AM P1.8, BA P1.4) belonging to the genera *Halobacillus*, *Planococcus* and *Idiomarina* showed high proteolytic activities. In addition to the eight pectinolytic isolates belonging to the genus *Bacillus*, two strains (AM P2.5 and CB N P1.4) related to *Psychrobacter* sp. and *Halomonas* sp. were able to degrade and use pectin as a carbon source. Moreover, an actinobacterial strain (BSL P1.X2) closely affiliated to species of the genus *Nocardiopsis* exhibited high xylanolytic and lipolytic activities (Figure 5).

Among the extremely halophilic archaea, the most promising strain in terms of extremozymes production was related to *Haloterrigena turkmenica*. This isolate (MM N EP2.5) had remarkable amylolytic and cellulolytic activities, albeit its main disadvantage was the slow growth rate. Furthermore, two isolates (AM N EP2.14 and BA N EP3.1) related to *Halostagnicola larsenii* were good amylase producers. The finding of these two extremely halophilic isolates in the brackish lakes AM and BA, but not in the saline and hypersaline environments investigated, was quite unexpected. One possible explanation for this finding could be related to the avian carriers [63]. In this respect, extremely halophilic archaea from hypersaline habitats could have been carried on bird feathers and distributed during migration to less saline environments. This hypothesis could also be supported by other previous studies that reported the capacity of halophilic archaea to remain viable at low salinities [36,64].

## 5. Conclusions

Salt-tolerant enzymes produced by halophilic and halotolerant microorganisms have been proposed as more efficient alternatives to mesophilic counterparts for catalyzing various industrial reactions carried out under high salinity conditions [8,9,10]. For instance, some halophilic hydrolases such as amylase, cellulase, xylanase and lipase may be used for the breakdown of different kinds of non-food biomasses and the production of biofuels [16]. Furthermore, such extremozymes may be useful in the bioremediation of hypersaline environments contaminated with organic compounds [18,19] and in the biocleaning of mural paintings [65].

Considering the biotechnological importance of halophilic enzymes and the current need for more efficient producers of such biocatalysts, the present paper reported the isolation of different environmental microbial taxa able to synthesize six extracellular hydrolases (i.e., protease, lipase, amylase, cellulase, xylanase and pectinase). According to our results, the best hydrolytic activities were observed in halotolerant species belonging to the genus *Bacillus*. In addition, some extremely halophilic archaea closely related to members of the genera *Haloterrigena* and *Halostagnicola* showed promising amylolytic and cellulolytic activities for biotechnological applications. Further investigations should be directed particularly towards the purification and the biochemical characterization of these enzymes. Moreover, the cloning of the corresponding genes could be a good approach for the efficient production of halophilic enzymes originating from slow-growing species.

## Figures and Tables

**Table 1:** Physicochemical properties of the sampled lakes ^1,2^.

Lake	pH	T (°C)	DO (mg·L^−1^)	ORP (mV)	EC (mS·cm^−1^)	Salinity (g·L^−1^)	Chloride (g·L^−1^)
AM	8.81 ± 0.3	25.49 ± 0.03	11.54 ± 1.29	12.67 ± 2.27	19.05 ± 0.25	11.31 ± 0.15	3.54 ± 0.07
BA	10.16 ± 0.25	24.4 ± 0.0	3.86 ± 0.19	−158 ± 17.9	20.78 ± 0.18	12.36 ± 0.11	5.52 ± 0.07
CB	9.03 ± 0.23	29.96 ± 0.88	2.53 ± 1.81	−220 ± 58.1	52.8 ± 8.98	35.67 ± 5.85	17.91 ± 0.87
MM	10.15 ± 0.26	22.87 ± 0.09	2.12 ± 0.51	−94.7 ± 16.7	107.8 ± 0.2	>70 *	38.52 ± 0.54
BSL	8.05 ± 0.09	36.67 ± 2.75	0.71 ± 0.57	−339 ± 43.9	168.1 ± 4.9	>70 *	150.5 ± 5.9

^1^ Data are presented as mean ± standard deviation (*n* = 3). ^2^ Abbreviations: T, Temperature; DO, Dissolved Oxygen; ORP, Oxidation Reduction Potential; EC, Electrical Conductivity. * Values exceeded the detection limit of the measuring instrument.

## Supplementary Materials

The following are available online at https://www.mdpi.com/2076-2607/8/12/1903/s1, Figure S1: Neighbor-joining phylogenetic tree showing the relationship between *Firmicutes* isolates and closely related species based on 16S rRNA gene sequence comparison; Figure S2: Neighbor-joining phylogenetic tree showing the relationship between *Proteobacteria*-*Actinobacteria* isolates and closely related species based on 16S rRNA gene sequence comparison; Figure S3: Neighbor-joining phylogenetic tree showing the relationship between *Archaeal* isolates and closely related species based on 16S rRNA gene sequence comparison.

## References

[1] Oren A. (2002). Diversity of Halophilic Microorganisms: Environments, Phylogeny, Physiology, and Applications. J. Ind. Microbiol. Biotechnol..

[2] McGenity T.J., Oren A., Bell E.M. (2012). Hypersaline Environments. Life at Extremes: Environments, Organisms and Strategies for Survival.

[3] Oren A., Hurst C.J. (2016). Life in Hypersaline Environments. Their World: A Diversity of Microbial Environments, Advances in Environmental Microbiology.

[4] Ventosa A., Márquez M.C., Sánchez-Porro C., de la Haba R.R., Vreeland R.H. (2012). Taxonomy of Halophilic Archaea and Bacteria. Advances in Understanding the Biology of Halophilic Microorganisms.

[5] Gunde-Cimerman N., Plemenitaš A., Oren A. (2018). Strategies of Adaptation of Microorganisms of the Three Domains of Life to High Salt Concentrations. FEMS Microbiol. Rev..

[6] Ventosa A., de la Haba R.R., Sánchez-Porro C., Papke R.T. (2015). Microbial Diversity of Hypersaline Environments: A Metagenomic Approach. Curr. Opin. Microbiol..

[7] Oren A. (2015). Halophilic Microbial Communities and Their Environments. Curr. Opin. Biotechnol..

[8] Ventosa A., Nieto J.J. (1995). Biotechnological Applications and Potentialities of Halophilic Microorganisms. World J. Microbiol. Biotechnol..

[9] Oren A. (2010). Industrial and Environmental Applications of Halophilic Microorganisms. Environ. Technol..

[10] Liu C., Baffoe D.K., Zhan Y., Zhang M., Li Y., Zhang G. (2019). Halophile, an Essential Platform for Bioproduction. J. Microbiol. Methods.

[11] Yin J., Chen J.-C., Wu Q., Chen G.-Q. (2015). Halophiles, coming stars for industrial biotechnology. Biotechnol. Adv..

[12] Giani M., Garbayo I., Vilchez C., Martinez-Espinosa R.M. (2019). Haloarchaeal Carotenoids: Healthy Novel Compounds from Extreme Environments. Mar. Drugs.

[13] Corral P., Amoozegar M.A., Ventosa A. (2020). Halophiles and Their Biomolecules: Recent Advances and Future Applications in Biomedicine. Mar. Drugs.

[14] Tadeo X., López-Méndez B., Trigueros T., Laín A., Castaño D., Millet O. (2009). Structural Basis for the Aminoacid Composition of Proteins from Halophilic Archea. PLoS Biol..

[15] Siglioccolo A., Paiardini A., Piscitelli M., Pascarella S. (2011). Structural Adaptation of Extreme Halophilic Proteins through Decrease of Conserved Hydrophobic Contact Surface. BMC Struct. Biol..

[16] Amoozegar M.A., Safarpour A., Noghabi K.A., Bakhtiary T., Ventosa A. (2019). Halophiles and Their Vast Potential in Biofuel Production. Front. Microbiol..

[17] Cai Z.-W., Ge H.-H., Yi Z.-W., Zeng R.-Y., Zhang G.-Y. (2018). Characterization of a Novel Psychrophilic and Halophilic β-1,3-Xylanase from Deep-Sea Bacterium, *Flammeovirga pacifica* Strain WPAGA1. Int. J. Biol. Macromol..

[18] Le Borgne S., Paniagua D., Vazquez-Duhalt R. (2008). Biodegradation of Organic Pollutants by Halophilic Bacteria and Archaea. J. Mol. Microbiol. Biotechnol..

[19] Fathepure B.Z. (2014). Recent Studies in Microbial Degradation of Petroleum Hydrocarbons in Hypersaline Environments. Front. Microbiol..

[20] Bulgareanu V.A.C. (1993). The Protection and Management of Saline Lakes of Therapeutic Value in Romania. Int. J. Salt Lake Res..

[21] Gastescu P. (1971). Lacurile din Romania—Limnologie Regionala.

[22] Radulescu C., Dulama I.D., Stihi C., Ionita I., Chilian A., Necula C., Chelarescu E.D. (2014). Determination of Heavy Metal Levels in Water and Therapeutic Mud by Atomic Absorption Spectrometry. Rom. J. Phys..

[23] Plesoianu D., Diaconescu V. (2016). Amara, a Spa with an Extraordinary Natural Potential. Sci. Pap. Ser. Manag. Econ. Eng. Agric. Rural Dev..

[24] Gheorghievici L., Gheorghievici G., Tanase I. (2015). The Phytoplankton Composition Features of Five Romanian Pelogenous Ecosystems. Environ. Eng. Manag. J..

[25] Chelarescu E.D., Radulescu C., Stihi C., Bretcan P., Tanislav D., Dulama I.D., Stirbescu R.M., Teodorescu S., Bucurica I.A., Andrei R. (2017). Analysis of Elements in Lake Sediment Samples by PIXE Spectrometry. Nucl. Instrum. Methods Phys. Res. B.

[26] Voicu V., Burtea M.C., Mocanu V., Dumitru S. (2017). Seasonal Variation of Water Mineralisation Degree from Movila Miresii Lake and its Influence on Neighbouring Soils. J. Environ. Prot. Ecol..

[27] Radulescu C., Bucurica I.A., Bretcan P., Chelarescu E.D., Tanislav D., Dulama I.D., Stirbescu R.M., Teodorescu S. (2019). Complex Investigation of Unconsolidated Sediments of Romanian Plain Salt Lake. Rom. J. Phys..

[28] Moldoveanu M., Florescu L., Parpala L., Cojoc R., Enache M. (2015). Romanian Salt Lakes: Some Physical-Chemical Features and Composition of Biological Communities. Muz. Olten. Craiova Olten. Stud. şi comunicări Ştiinţele Nat..

[29] Axinte O., Badescu I.S., Stroe C., Neacsu V., Bulgariu L., Bulgariu D. (2015). Evolution of Trophic Parameters from Amara Lake. Environ. Eng. Manag. J..

[30] Neagu S., Enache M., Cojoc R. (2014). Extracellular Hydrolytic Activities of Halophilic Microorganisms Isolated from Balta Alba Salt Lake. Rom. Biotechnol. Lett..

[31] Ruginescu R.M., Cojoc R., Enache M., Lazar V. (2018). Preliminary Characterization of a Cellulase Producing Bacterial Strain Isolated from a Romanian Hypersaline Lake. J. Environ. Prot..

[32] Bulzu P.-A., Andrei A.-Ş., Salcher M.M., Mehrshad M., Inoue K., Kandori H., Beja O., Ghai R., Banciu H.L. (2019). Casting Light on Asgardarchaeota Metabolism in a Sunlit Microoxic Niche. Nat. Microbiol..

[33] Hauser B.A., Hauser B.A. (2002). Chloride. Drinking Water Chemistry: A Laboratory Manual.

[34] Ventosa A., Quesada E., Rodriguez-Valera F., Ruiz-Berraquero F., Ramos-Cormenzana A. (1982). Numerical Taxonomy of Moderately Halophilic Gram-Negative Rods. Microbiology.

[35] Chen S., Xu Y., Sun S., Chen F., Liu J. (2019). *Halalkalicoccus subterraneus* Sp. Nov., an Extremely Halophilic Archaeon Isolated from a Subterranean Halite Deposit. Antonie van Leeuwenhoek.

[36] Vauclare P., Natali F., Kleman J.P., Zaccai G., Franzetti B. (2020). Surviving Salt Fluctuations: Stress and Recovery in *Halobacterium salinarum*, an Extreme Halophilic Archaeon. Sci. Rep..

[37] Sanders E.R. (2012). Aseptic Laboratory Techniques: Plating Methods. J. Vis. Exp..

[38] Kushner D.J., Vreeland R.H., Hochstein L.I. (1992). Growth and Nutrition of Halophilic Bacteria. The Biology of Halophilic Bacteria.

[39] Rohban R., Amoozegar M.A., Ventosa A. (2009). Screening and Isolation of Halophilic Bacteria Producing Extracellular Hydrolyses from Howz Soltan Lake, Iran. J. Ind. Microbiol. Biotechnol..

[40] Menasria T., Aguilera M., Hocine H., Benammar L., Ayachi A., Si Bachir A., Dekak A., Monteoliva-Sánchez M. (2018). Diversity and Bioprospecting of Extremely Halophilic Archaea Isolated from Algerian Arid and Semi-Arid Wetland Ecosystems for Halophilic-Active Hydrolytic Enzymes. Microbiol. Res..

[41] Enache M., Itoh T., Kamekura M., Teodosiu G., Dumitru L. (2007). *Haloferax prahovense* Sp. Nov., an Extremely Halophilic Archaeon Isolated from a Romanian Salt Lake. Int. J. Syst. Evol. Microbiol..

[42] Latorre J.D., Hernandez-Velasco X., Wolfenden R.E., Vicente J.L., Wolfenden A.D., Menconi A., Bielke L.R., Hargis B.M., Tellez G. (2016). Evaluation and Selection of *Bacillus* Species Based on Enzyme Production, Antimicrobial Activity, and Biofilm Synthesis as Direct-Fed Microbial Candidates for Poultry. Front. Vet. Sci..

[43] Fredriksson N.J., Hermansson M., Wilén B.-M. (2013). The Choice of PCR Primers Has Great Impact on Assessments of Bacterial Community Diversity and Dynamics in a Wastewater Treatment Plant. PLoS ONE.

[44] Basic Local Alignment Search Tool. https://blast.ncbi.nlm.nih.gov/Blast.cgi.

[45] Tamura K., Nei M. (1993). Estimation of the number of nucleotide substitutions in the control region of mitochondrial DNA in humans and chimpanzees. Mol. Biol. Evol..

[46] van der Lee G.H., Kraak M.H.S., Verdonschot R.C.M., Vonk J.A., Verdonschot P.F.M. (2017). Oxygen Drives Benthic-Pelagic Decomposition Pathways in Shallow Wetlands. Sci. Rep..

[47] Oren A., Meng F.-W. (2019). ‘Red—The magic color for solar salt production’—But since When?. FEMS Microbiol. Lett..

[48] Sánchez-Porro C., Martín S., Mellado E., Ventosa A. (2003). Diversity of moderately halophilic bacteria producing extracellular hydrolytic enzymes. J. Appl. Microbiol..

[49] Al-Rubaye M.T.S., Al-Musawi M.H.J., Fakhari J., Hosseini M. (2017). Screening and Characterization of Halophilic Bacteria With Industrial Enzymes from Salt Lake Razazah, Karbala, Iraq. Biosci. Biotechnol. Res. Asia.

[50] Quadri I., Hassani I.I., l’Haridon S., Chalopin M., Hacène H., Jebbar M. (2016). Characterization and Antimicrobial Potential of Extremely Halophilic Archaea Isolated from Hypersaline Environments of the Algerian Sahara. Microbiol. Res..

[51] Orwa P., Mugambi G., Wekesa V., Mwirichia R. (2020). Isolation of Haloalkaliphilic Fungi from Lake Magadi in Kenya. Heliyon.

[52] Kaitouni L.B.D., Anissi J., Sendide K., El Hassouni M. (2020). Diversity of Hydrolase-Producing Halophilic Bacteria and Evaluation of Their Enzymatic Activities in Submerged Cultures. Ann. Microbiol..

[53] Stewart E.J. (2012). Growing Unculturable Bacteria. J. Bacteriol..

[54] Hashemzahi A., Makhdoumi A., Asoodeh A. (2020). Culturable Diversity and Enzyme Production Survey of Halophilic Prokaryotes from a Solar Saltern on the Shore of the Oman Sea. J. Genet. Resour..

[55] Daoud Z., Sura M., Roula A.-M. (2013). Pectin Shows Antibacterial Activity against *Helicobacter pylori*. Adv. Biosci. Biotechnol..

[56] Jindal M., Kumar V., Rana V., Tiwary A.K. (2013). Aegle Marmelos Fruit Pectin for Food and Pharmaceuticals: Physico-Chemical, Rheological and Functional Performance. Carbohydr. Polym..

[57] Zofou D., Shu G.L., Foba-Tendo J., Tabouguia M.O., Assob J.-C.N. (2019). In Vitro and In Vivo Anti-Salmonella Evaluation of Pectin Extracts and Hydrolysates from “Cas Mango” (Spondias Dulcis). Evid. Based Complement. Altern. Med..

[58] Dang H., Zhu H., Wang J., Li T. (2009). Extracellular Hydrolytic Enzyme Screening of Culturable Heterotrophic Bacteria from Deep-Sea Sediments of the Southern Okinawa Trough. World J. Microbiol. Biotechnol..

[59] Chamekh R., Deniel F., Donot C., Jany J.-L., Nodet P., Belabid L. (2019). Isolation, Identification and Enzymatic Activity of Halotolerant and Halophilic Fungi from the Great Sebkha of Oran in Northwestern of Algeria. Mycobiology.

[60] Menasria T., Monteoliva-Sánchez M., Benammar L., Benhadj M., Ayachi A., Hacène H., Gonzalez-Paredes A., Aguilera M. (2019). Culturable Halophilic Bacteria Inhabiting Algerian Saline Ecosystems: A Source of Promising Features and Potentialities. World J. Microbiol. Biotechnol..

[61] Harwood C.R. (1992). *Bacillus subtilis* and Its Relatives: Molecular Biological and Industrial Workhorses. Trends Biotechnol..

[62] Fujinami S., Fujisawa M. (2010). Industrial Applications of Alkaliphiles and Their Enzymes—Past, Present and Future. Environ. Technol..

[63] Kemp B.L., Tabish E.M., Wolford A.J., Jones D.L., Butler J.K., Baxter B.K. (2018). The Biogeography of Great Salt Lake Halophilic Archaea: Testing the Hypothesis of Avian Mechanical Carriers. Diversity.

[64] Purdy K.J., Cresswell-Maynard T.D., Nedwell D.B., McGenity T.J., Grant W.D., Timmis K.N., Embley T.M. (2004). Isolation of Haloarchaea That Grow at Low Salinities. Environ. Microbiol..

[65] Gomoiu I., Dumbravician M., Mohanu M., Enache M., Neagu S., Ruginescu R., Cojoc R. Cleaning of Mural Paintings and Mortars: Review.

