# Supplementary material for: Bioprospecting for Novel Halophilic and Halotolerant Sources of Hydrolytic Enzymes in Brackish, Saline and Hypersaline Lakes of Romania
Source: Microorganisms. 2020 Nov 30;8(12):1903. doi: 10.3390/microorganisms8121903 (PMC7760675; doi:10.3390/microorganisms8121903)
Supplement: Supplementary file 1 [file microorganisms-08-01903-s001.pdf]

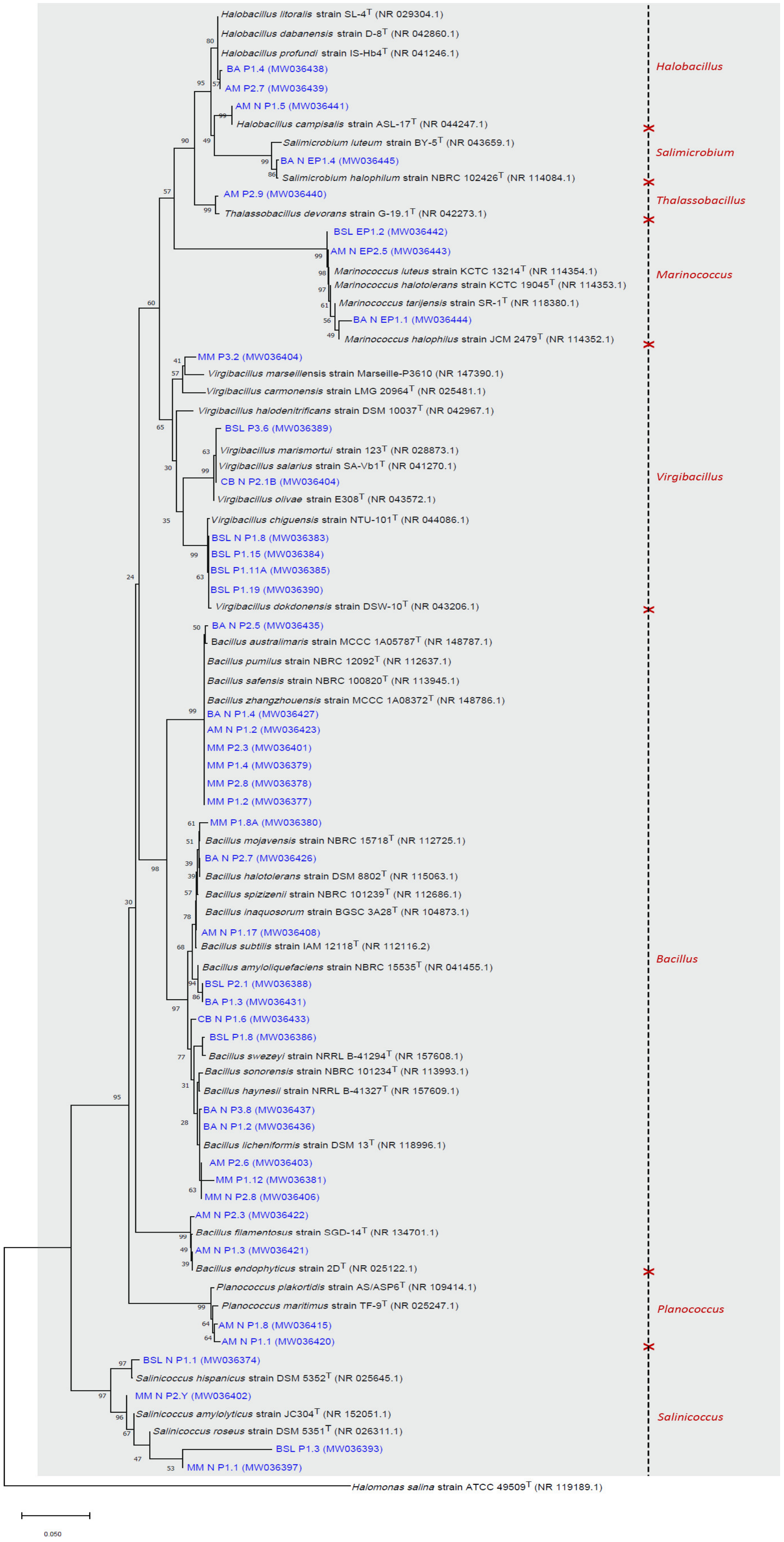

Figure S1. Neighbor-joining phylogenetic tree showing the relationship between *Firmicutes* isolates and closely related species based on 16S rRNA gene sequence comparison. Accession numbers are shown in parentheses. Bootstrap values based on 10000 replicates are shown. Bar, 0.05 substitutions per nucleotide position. The sequence of *Halomonas salina* was used as the outgroup.

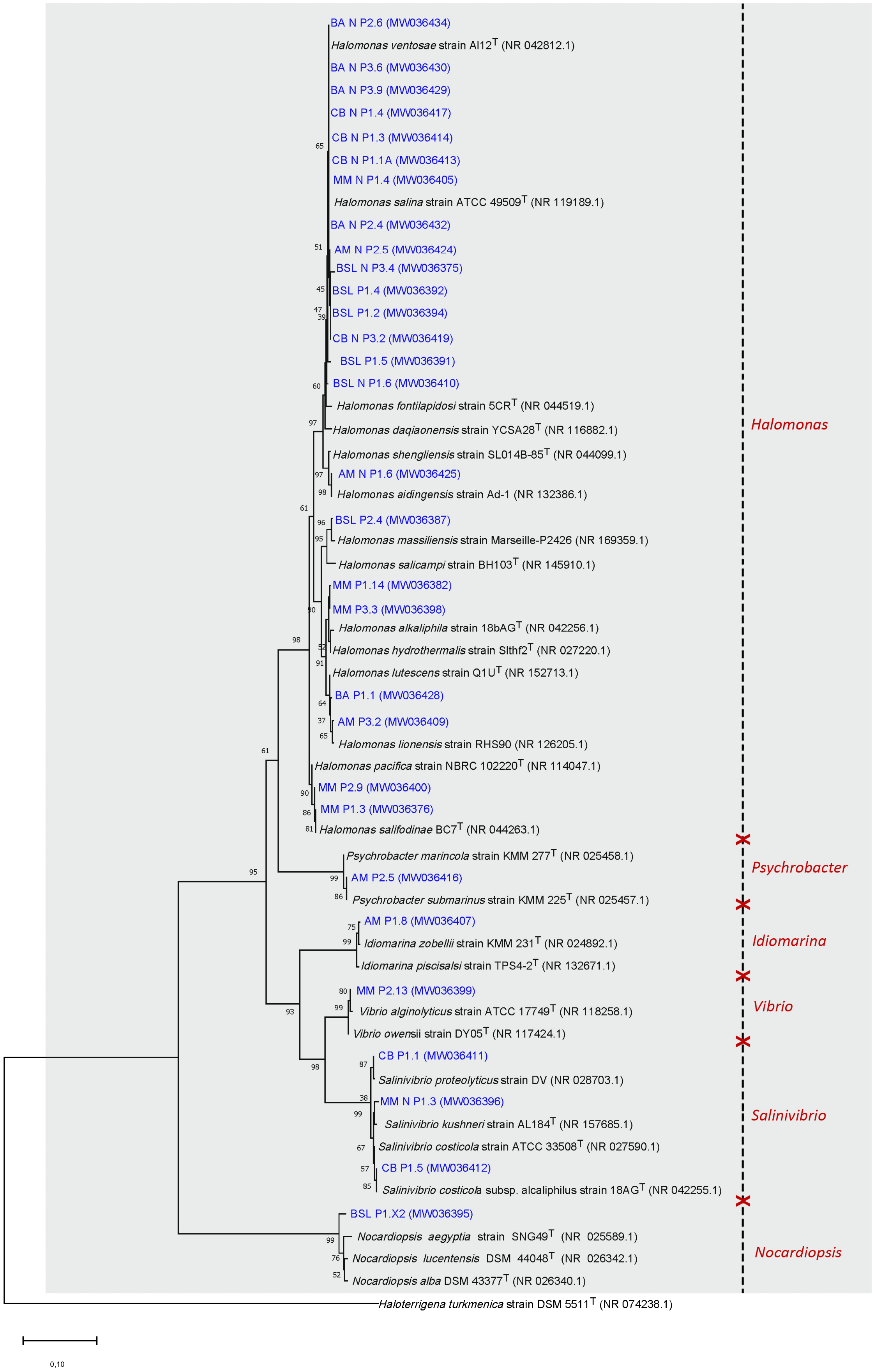

Figure S2. Neighbor-joining phylogenetic tree showing the relationship between *Proteobacteria-Actinobacteria* isolates and closely related species based on 16S rRNA gene sequence comparison. Accession numbers are shown in parentheses. Bootstrap values based on 10000 replicates are shown. Bar, 0.1 substitutions per nucleotide position. The sequence of *Haloterrigena turkmenica* was used as the outgroup.

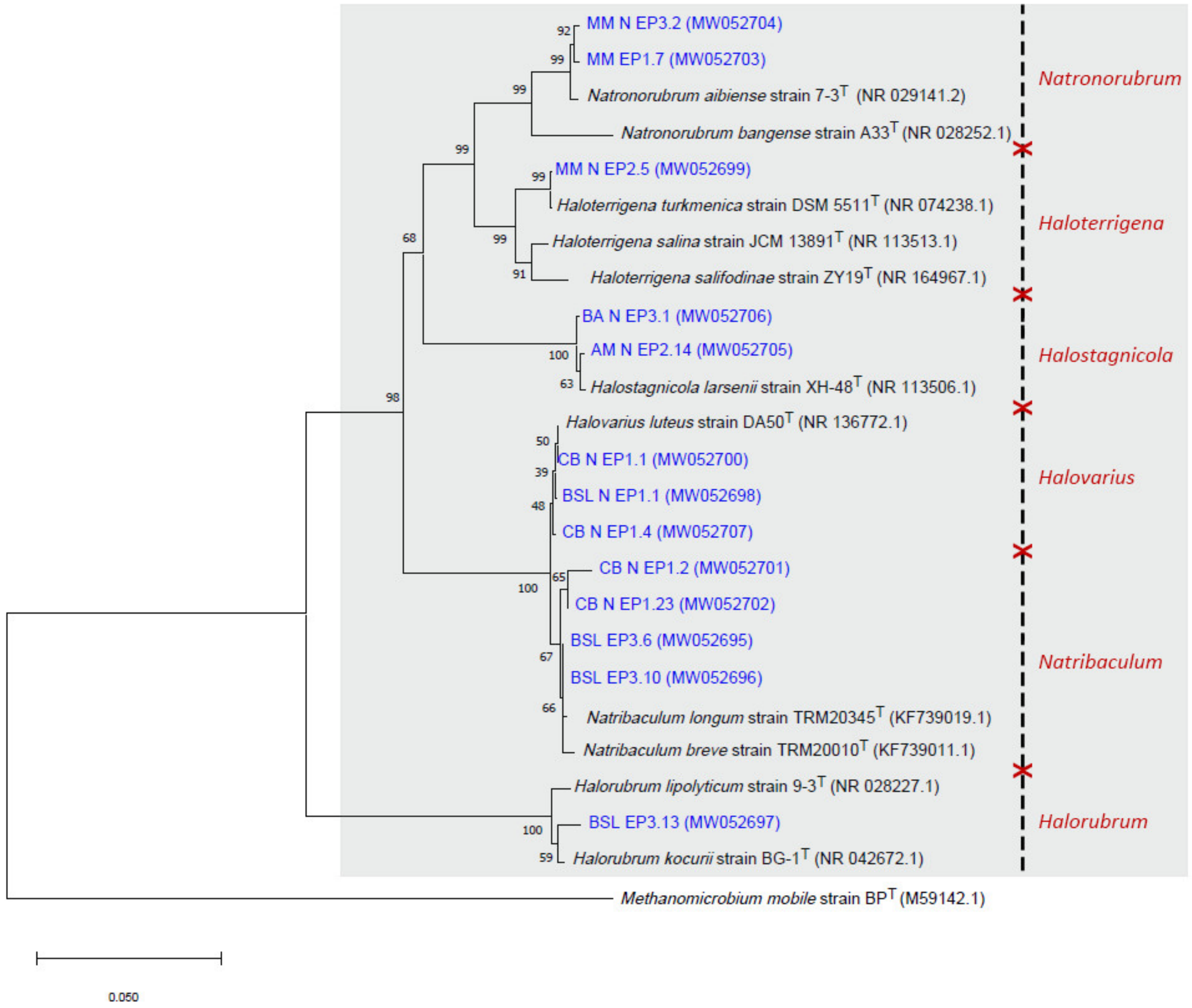

Figure S3. Neighbor-joining phylogenetic tree showing the relationship between *Archaeal* isolates and closely related species based on 16S rRNA gene sequence comparison. Accession numbers are shown in parentheses. Bootstrap values based on 10000 replicates are shown. Bar, 0.05 substitutions per nucleotide position. The sequence of *Methanomicrobium mobile* was used as the outgroup.
